# Non‐invasive imaging of functional pancreatic islet beta‐cell mass in people with type 1 diabetes mellitus

**DOI:** 10.1111/dme.15111

**Published:** 2023-04-21

**Authors:** Shruti S. Joshi, Trisha Singh, Lucy E. Kershaw, Fraser W. Gibb, Marc R. Dweck, Michelle Williams, Iskandar Idris, Scott Semple, Shareen Forbes, David E. Newby, Rebecca M. Reynolds

**Affiliations:** ^1^ British Heart Foundation Centre for Cardiovascular Science University of Edinburgh Edinburgh UK; ^2^ Edinburgh Imaging University of Edinburgh Edinburgh UK; ^3^ Edinburgh Centre for Endocrinology NHS Lothian Edinburgh UK; ^4^ Department of Radiology NHS Lothian Edinburgh UK; ^5^ Department of Endocrinology University of Nottingham Nottingham UK

**Keywords:** magnetic resonance imaging, pancreatic beta‐cells, type 1 diabetes mellitus

## Abstract

**Aims:**

To investigate whether manganese‐enhanced magnetic resonance imaging can assess functional pancreatic beta‐cell mass in people with type 1 diabetes mellitus.

**Methods:**

In a prospective case–control study, 20 people with type 1 diabetes mellitus (10 with low (≥50 pmol/L) and 10 with very low (<50 pmol/L) C‐peptide concentrations) and 15 healthy volunteers underwent manganese‐enhanced magnetic resonance imaging of the pancreas following an oral glucose load. Scan‐rescan reproducibility was performed in 10 participants.

**Results:**

Mean pancreatic manganese uptake was 31 ± 6 mL/100 g of tissue/min in healthy volunteers (median 32 [interquartile range 23–36] years, 6 women), falling to 23 ± 4 and 13 ± 5 mL/100 g of tissue/min (*p* ≤ 0.002 for both) in people with type1 diabetes mellitus (52 [44–61] years, 6 women) and low or very low plasma C‐peptide concentrations respectively. Pancreatic manganese uptake correlated strongly with plasma C‐peptide concentrations in people with type1 diabetes mellitus (*r* = 0.73, *p* < 0.001) but not in healthy volunteers (*r* = −0.054, *p* = 0.880). There were no statistically significant correlations between manganese uptake and age, body‐mass index, or glycated haemoglobin. There was strong intra‐observer (mean difference: 0.31 (limits of agreement −1.42 to 2.05) mL/100 g of tissue/min; intra‐class correlation, ICC = 0.99), inter‐observer (−1.23 (−5.74 to 3.27) mL/100 g of tissue/min; ICC = 0.85) and scan‐rescan (−0.72 (−2.9 to 1.6) mL/100 g of tissue/min; ICC = 0.96) agreement for pancreatic manganese uptake.

**Conclusions:**

Manganese‐enhanced magnetic resonance imaging provides a potential reproducible non‐invasive measure of functional beta‐cell mass in people with type 1 diabetes mellitus. This holds major promise for investigating type 1 diabetes, monitoring disease progression and assessing novel immunomodulatory interventions.

**What's new?**

- Type 1 diabetes mellitus is characterised by autoimmune destruction of pancreatic beta‐cells resulting in insulin deficiency. Evaluation of novel immunomodulatory therapies to prevent beta‐cell destruction requires reliable methods to measure functional beta‐cell mass which are currently unavailable.
- The current study has demonstrated, for the first time, that manganese‐enhanced magnetic resonance imaging can provide a potential non‐invasive imaging technique to assess the functional beta‐cell mass in people with type 1 diabetes mellitus.
- This has important implications for the investigation of the pathophysiology of diabetes, the monitoring of disease progression and the assessment of novel beta‐cell preserving therapeutic interventions.

## 1 INTRODUCTION

Type 1 diabetes mellitus is characterised by autoimmune destruction of pancreatic beta‐cells resulting in insulin deficiency.^1^ The destruction of beta‐cells precedes the clinical manifestation of the disease and impaired insulin secretion can be detected several years prior to the onset of hyperglycaemia.^2^ Beta‐cell mass is reduced by 80%–90% at the time of diagnosis^1^, ^3^ and the degree of beta‐cell dysfunction often exceeds beta‐cell loss,^4^ indicating additional functional impairment in insulin secretion. Both beta‐cell mass and function further decline over time, but are not always reflected in plasma C‐peptide concentrations.^5^, ^6^ It is now recognised that there are micro‐secretors of insulin and that preservation of insulin secretion in these cases is associated with several physiological advantages, such as reduction in hypoglycaemic episodes, improved quality of life and reduced hospitalisations.^7^, ^8^ This provides a rationale for novel therapeutic strategies aimed at restoring or at least preventing further loss of beta‐cell mass in type 1 diabetes mellitus.

Evaluation of novel immunomodulatory therapies to prevent beta‐cell destruction requires reliable methods to measure beta‐cell function. Although plasma C‐peptide concentrations can be measured with high sensitivity assays, it does not reflect the functional numbers of beta cells present.^9^ Therefore, novel methods to evaluate functional beta cell mass would be invaluable for understanding the pathophysiology of type 1 diabetes mellitus, tracking disease progression, and assessing treatment response. For effective in vivo beta‐cell imaging, contrast media need to be sufficiently selective for beta‐cells, to provide a stable high‐intensity signal, and to reflect the functional state of beta‐cells.^10^ An example includes the use of gadolinium‐based zinc‐sensitive agents which exploit the fact that zinc ions are secreted along with insulin from the pancreatic beta cells. Translation of this approach into human subjects has yet to be established.^11^ An alternative new and readily translatable approach is manganese‐enhanced magnetic resonance imaging.

Traditional magnetic resonance contrast media incorporate paramagnetic metal ions that are irreversibly chelated to provide tissue contrast enhancement when they enter the extracellular space. They do not cross intact cell membranes and are unable to provide intracellular contrast. Mangafodipir represents an alternative approach where manganese is partially chelated by dipyridoxyl diphosphate and circulates within a protein bound complex. After intravenous administration, mangafodipir releases manganese ions into plasma by dephosphorylation and transmetallation with zinc thereby allowing rapid intracellular manganese uptake and renal clearance. This approach mitigates the toxicity seen with non‐chelated forms of manganese‐based contrast media.^12^ Manganese has paramagnetic properties which shorten T1 relaxation and free dissociated manganese ions are taken up into pancreatic beta‐cells during insulin secretion via voltage‐gated calcium channels, serving as an intracellular contrast agent.^13^ Manganese and calcium ions compete during insulin secretion, thereby leading to pancreatic enhancement with manganese contrast which is dependent on the functional beta cell mass. The specificity of this technique for pancreatic beta cells has been demonstrated in various pre‐clinical studies using pharmacological treatments, such as nifedipine, tolbutamide and diazoxide, that activate or inactivate the voltage‐gated calcium channels in the pancreatic beta cells.^14^, ^15^

In this proof‐of‐concept study, we investigated whether manganese‐enhanced magnetic resonance imaging can be used as a non‐invasive and reproducible measure of pancreatic beta‐cell function in people with type 1 diabetes mellitus. Specifically, we hypothesised that pancreatic manganese uptake as assessed by T1 mapping will be reduced in people with type 1 diabetes mellitus and that this will be proportional to plasma C‐peptide concentrations.

## 2 RESEARCH DESIGN AND METHODS

### 2.1 Study design

We conducted a prospective proof‐of‐concept investigational study (NCT05298735) in accordance with the Declaration of Helsinki, a favourable ethical opinion from the research ethics committee (20/WM/0304) and the written informed consent of all participants.

### 2.2 Study participants

People with a confirmed diagnosis of type 1 diabetes mellitus, aged ≥18 years were identified and approached through the Edinburgh Centre for Endocrinology and Diabetes, Royal Infirmary of Edinburgh, Edinburgh, UK. We specifically recruited 10 people with low (>50 pmol/L) and 10 people with very low (<50 pmol/L) baseline plasma C‐peptide concentrations. Healthy volunteers were recruited via advertisement with inclusion criteria of age ≥ 18 years, no regular medication, and no known clinically significant medical condition. Exclusion criteria for all study participants included renal failure (estimated glomerular filtration rate < 30 mL/min/1.73 m^2^), an inability to consent, contraindications to magnetic resonance imaging, symptoms suggestive of coronavirus disease 2019 (COVID‐19), and pregnancy or breast‐feeding.

All participants had an assessment of clinical haematology and biochemistry including full blood count, liver and renal function, and glycated haemoglobin. C‐peptide concentrations were re‐measured after a standardised oral glucose load on the day of the scan (see below). Plasma C‐peptide concentrations were measured using an immunoassay (ARCHITECT C‐peptide assay, Abbott). Internal assay validation demonstrated a coefficient of variation of 7% at 7 pmol/L and of 15% at 4 pmol/L.

### 2.3 Magnetic resonance imaging

Subjects fasted from midnight and, on the following morning, were given a standardised glucose load (125 mL of Fortisip compact, 2.4 Kcal/mL [Nutricia] supplement drink)^16^ half an hour prior to their imaging to stimulate pancreatic beta‐cell insulin secretion. People with type 1 diabetes mellitus withheld any quick‐acting insulin prior to the scan.

Magnetic resonance imaging was performed using a 3T scanner (MAGNETOM Skyrafit; Siemens Healthineers) with a dedicated 30‐channel body matrix coil. Manganese‐enhanced magnetic resonance imaging was performed using intravenous manganese dipyridoxyl diphosphate (5 μmol/kg (0.1 mL/kg) at 1 mL/min; Exova SL Pharma) administered over 10 min. Localisers, half‐Fourier single‐shot turbo spin‐echo (HASTE) and standard breath‐hold cine sequences of the heart were followed by T1 mapping sequences of the pancreas. Pancreatic T1 mapping was performed prior to contrast using a breath‐held modified Look‐Locker inversion recovery acquisition and every 2.5 min for 30 min after commencing the intravenous manganese dipyridoxyl diphosphate infusion.^17^ Pancreas and left ventricular blood pool T1 values were compared.

### 2.4 Image analysis

Magnetic resonance images were analysed offline using Circle CVI (Circle Cardiovascular Imaging, CVI42 v5.13.5). Quantitative analysis of manganese accumulation was performed by measuring T1 relaxation time in the region of interest drawn within the head, body and tail of the pancreas and compared with the left ventricular blood pool. T1 maps were generated using commercially available software (CVI42, Circle cardiovascular imaging) and applying a three‐parameter non‐linear curve fitting as described previously.^18^ Analysis was performed by experienced observers (SJ, TS).

### 2.5 Manganese kinetic modelling

Patlak et al. were the first to provide a graphical analysis of unidirectionality of transfer and of the influx constant, using brain uptake data.^19^ The multi‐timepoint approach produces information about the exchange rate of the compartments that rapidly and reversibly exchange with plasma. Moreover, this can be used to assess the rate constant of any type of irreversible process within any organ system. The Patlak two‐compartment model was used in this study to measure the rate of pancreatic manganese uptake. This assumes the influx of manganese ions from a reversible (*v*_e_, extracellular and vascular space) into a largely irreversible (*v*_i_, pancreatic beta cells during the imaging period) compartment. This apparent unidirectional influx constant (Ki) for the transfer of manganese from plasma to irreversible compartments *v*_i_, can be measured, using the equation below:

$$\frac{C_{t}\left(t\right)}{C_{a}\left(t\right)}=\mathrm{Ki}\frac{\int_{0}^{t}C_{t}\left(\tau \right)\mathrm{d\tau}}{C_{a}\left(t\right)}+v_{e},$$

where *C*_*t*_ and *C*_a_ are the manganese concentration in pancreatic tissue and blood pool (arterial input function) respectively. This formula is equivalent to the Patlak model and describes that if a contrast medium is irreversibly trapped in the tissue within the imaging period, the instantaneous tissue concentration (pancreatic T1) divided by the instantaneous arterial concentration (blood pool T1) plotted against the integrated arterial concentration divided by the instantaneous arterial concentration, will result in linearisation of the data.

### 2.6 Intra‐observer and Inter‐observer repeatability and scan‐rescan reproducibility

To assess intra‐observer repeatability, images from ten participants were analysed by the same observer (SJ) at two different time points, at least 8–12 weeks apart and in random order to minimise recall bias. Similarly, repeated measurements were made in 10 participants by two independent observers (SJ and TS) to assess inter‐observer repeatability. Ten participants also underwent repeat manganese‐enhanced magnetic resonance imaging to assess scan‐rescan reproducibility.

### 2.7 Statistical analysis

All statistical analyses were performed with R Studio (RStudio). Categorical baseline variables were presented as number (%) and compared using a Chi‐squared test. Continuous data were assessed for normality using the Shapiro test and presented as mean ± standard deviation or median [interquartile range]. Pancreatic manganese uptake measurements were compared using analysis of variance (ANOVA) and Tukey's HSD (honestly significant difference) post‐hoc test. Values were compared using paired or unpaired Student's *t*‐tests, Wilcoxon or Mann–Whitney tests and Kruskal–Wallis tests as appropriate. Correlation analyses between pancreatic manganese uptake and other parameters were performed using Spearman's rank correlation coefficient. Statistical significance was taken as two‐sided *p* < 0.05. Reproducibility and repeatability analyses were carried out using intra‐class correlation coefficient (ICC) and Bland Altmann analysis. A correlation coefficient with an integer value of 1.0 was considered as perfect correlation, between 0.9–1.0 very strong, between 0.70–0.90 strong, between 0.5–0.6 moderate and 0.1–0.4 weak.

## 3 RESULTS

### 3.1 Study population

We recruited 38 participants: 23 people with type 1 diabetes mellitus and 15 healthy volunteers. Three participants were withdrawn because of incomplete data acquisition due to claustrophobia (*n* = 2) and poor image quality due to body habitus (*n* = 1). People with type 1 diabetes mellitus were on average 20–25 years older than the healthy volunteers (Table 1). However, there were no correlations between pancreatic manganese uptake and age in either people with type 1 diabetes mellitus or healthy volunteers.

**TABLE 1:** Baseline characteristics of study participants.

	Healthy volunteers	People with type 1 diabetes mellitus and low plasma C‐peptide concentrations	People with type 1 diabetes mellitus and very low plasma C‐peptide concentrations
Number of participants	15	10	10
Age (years)	32 [22–36]	59 [40–70]	51 [46–56]
Sex (men:women)	9:6	7:3	7:3
Baseline plasma C‐peptide concentration (pmol/L)	794 [399–1591]	186 [124–387]	32 [0–61]
Body‐mass index (kg/m^2^)	24.5 [23.3–27.9]	26.0 [21.3–28.2]	27.4 [25.0–30.5]
Duration of diabetes mellitus (years)	N/A	9 [5–17]	25 [10–31]

*Note*: *n* (%), median [interquartile range].

### 3.2 Pancreatic magnetic resonance imaging

The native (pre‐contrast) pancreatic T1 values for people with type 1 diabetes mellitus were higher than those in healthy volunteers (950 [880–1000] vs. 820 [770–830] ms respectively, *p* < 0.001). During manganese dipyridoxyl diphosphate infusion, pancreatic T1 values demonstrated a rapid initial reduction followed by a gradual and sustained reduction. Median T1 values 30 min after manganese dipyridoxyl diphosphate infusion were higher in people with type 1 diabetes mellitus compared to healthy volunteers (595 [520–635] vs. 435 [415–430] ms respectively, *p* < 0.001).

**FIGURE 1:**
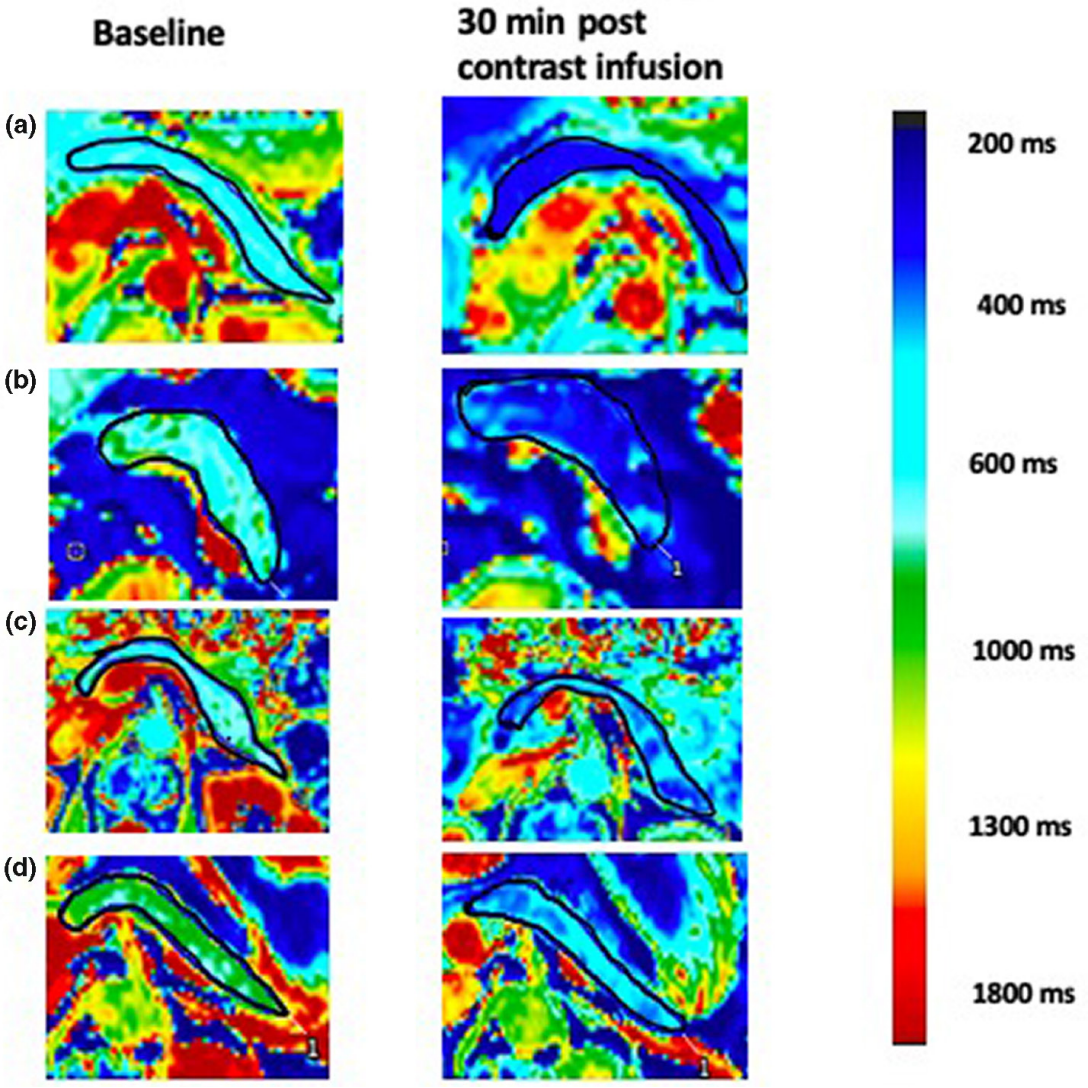
Example pancreatic T1 maps in study participants. Pancreas at baseline (left‐hand column) and 30 min after manganese contrast infusion (right‐hand column) in (a) healthy volunteer (plasma C‐peptide concentration > 500 pmol/L; Ki 35 mL/100 g of tissue/min), (b) individual with type 1 diabetes mellitus and low C‐peptide concentration (362 pmol/L; Ki 27 mL/100 g of tissue/min), (c) individual with type 1 diabetes mellitus and very low C‐peptide concentration (0 pmol/L; Ki 5 mL/100 g of tissue/min) and (d) individual with type 1 diabetes mellitus and very low C‐peptide concentration (32 mol/L; Ki 15 mL/100 g of tissue/min). Colours are assigned to different T1 values on a pixel‐by‐pixel basis. The colour scale shows T1 values corresponding to the different colours. T1 values represent the time constant for longitudinal relaxation time which is measured in milliseconds and is a standard measure for magnetic resonance imaging. Note the differences in the appearance of pancreas in participants with different C‐peptide concentrations. The areas with reduced enhancement post contrast represent reduced manganese uptake and likely areas of beta‐cell loss.

Compared to the healthy volunteers, participants with type 1 diabetes mellitus had visibly lower pancreatic enhancement 30 min after manganese contrast infusion. Moreover, participants with very low plasma C‐peptide concentrations had lower pancreatic enhancement compared with participants with low plasma C‐peptide concentrations (Figure 1). There were no regional differences in enhancement within the pancreatic tissue (head vs. body vs. tail).

**FIGURE 2:**
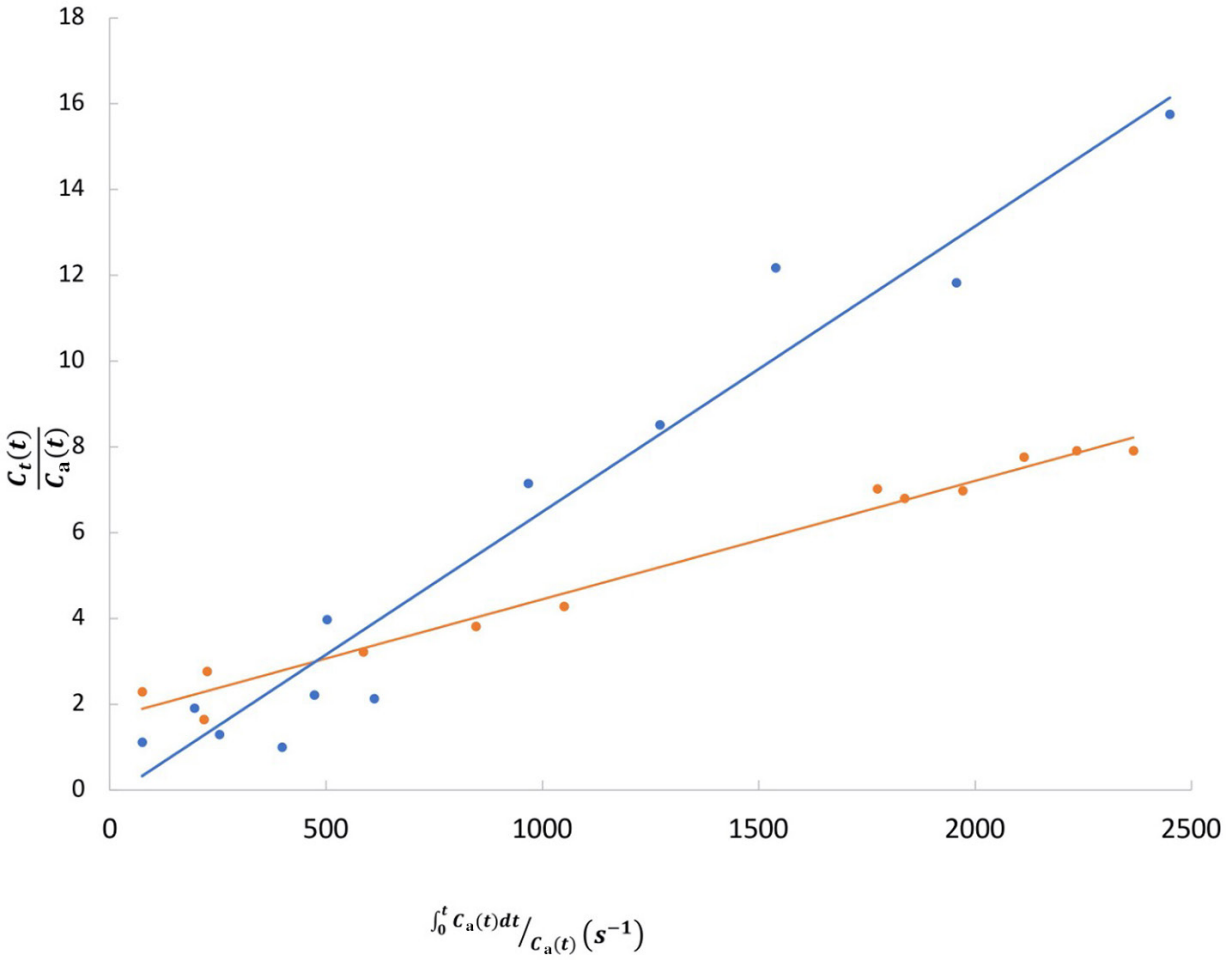
Patlak (Kinetic modelling) plots for a healthy volunteer (blue) and a participant with type 1 diabetes mellitus (orange). The gradient of the fitted straight line is equal to Ki, the manganese uptake constant. *C*_*t*_ = Manganese concentration in the pancreas, *C*_a_ = arterial Manganese concentration and *t* = time.

**FIGURE 3:**
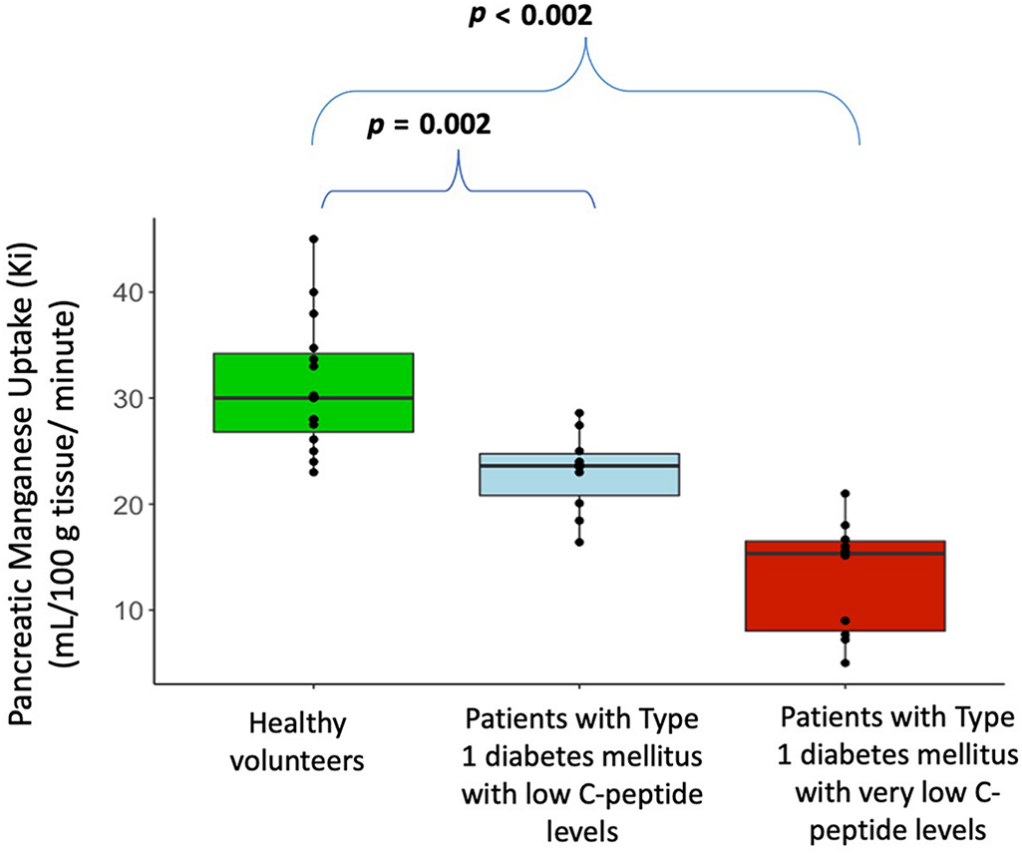
Pancreatic manganese uptake (Ki) in participant cohorts. Mean Ki was higher in healthy volunteers (31 ± 6 mL/100 g of tissue/min) compared to people with type 1 diabetes mellitus and low (23 ± 4 mL/100 g of tissue/min) or very low (13 ± 5 mL/100 g of tissue/min) plasma C‐peptide concentrations. ANOVA with Tukey's post hoc test *p* ≤ 0.002 for both versus healthy volunteers.

Kinetic modelling demonstrated a lower rate of pancreatic manganese uptake in participants with type 1 diabetes mellitus compared with healthy volunteers (Figure 2). Mean pancreatic manganese uptake (Ki) was 31 ± 6 mL/100 g of tissue/min in healthy volunteers falling to 23 ± 4 and 13 ± 5 mL/100 g of tissue/min in people with type 1 diabetes mellitus with low and very low C‐peptide concentrations respectively (*p* ≤ 0.002 for both vs. healthy volunteers; Figure 3). Pancreatic manganese uptake was strongly correlated with both baseline plasma C‐peptide and C‐peptide concentrations after a glucose load in people with type 1 diabetes mellitus (*r* = 0.73, *p* < 0.001 and *r* = 0.71, *p* = 0.007 respectively) but not in healthy volunteers (*r* = −0.054, *p* = 0.880; Figure S1). There were no statistically significant correlations between age and pancreatic Ki for either people with type 1 diabetes mellitus (*r* = −0.03, *p* = 0.894) or healthy volunteers (*r* = 0.31, *p* = 0.255). Similarly, there were no statistically significant correlations between the pancreas Ki and body‐mass index or glycated haemoglobin for people with type 1 diabetes mellitus.

### 3.3 Repeatability and reproducibility

#### 3.3.1 Intra‐observer repeatability

There were no differences in repeated measurements of pancreatic native T1 values (833 [778–848] vs. 824 [765–843] ms), post‐contrast T1 values (437 [426–436] vs. 435 [419–451] ms) and pancreatic manganese uptake (Ki, 30 [29–34] vs. 28 [26–33] mL/100 g of tissue/min). There were strong correlations between the paired pancreatic native T1 values (mean difference: −12.0 (limits of agreement: −10.8 to 34.8) ms, ICC = 0.93), post‐contrast T1 values (mean difference: −2.5 (limits of agreement: −31.4 to 26.4) ms, ICC = 0.87) and pancreatic manganese uptake (mean difference 0.31 (limits of agreement −1.42 to 2.05) mL/100 g of tissue/min; ICC = 0.99).

#### 3.3.2 Inter‐observer repeatability

There were no differences in repeated measurements of pancreatic native T1 values (833 [778–848] vs. 818 [763–837] ms), post‐contrast T1 values (438 [427–452] vs. 435 [414–450] ms) and pancreatic manganese uptake (30 [28–34] vs. 33 [29–35] mL/100 g tissue/min). There were strong correlations and good limits of agreement between the paired pancreatic native T1 values (mean difference: 25.5 (limits of agreement 0.98 to 50) ms; ICC = 0.83), post‐contrast T1 values (mean difference: 11.3 (limits of agreement: −10.5 to 33.1) ms; ICC = 0.80) and pancreatic manganese uptake, Ki (mean difference − 1.23 (limits of agreement −5.74 to 3.27) mL/100 g of tissue/min; ICC = 0.85).

#### 3.3.3 Scan‐rescan reproducibility

Repeated scans were performed 88 [62–124] days apart. There were no differences between native T1 (801 [785–826] vs. 806 [757–823] ms), post‐contrast T1 (441 [426–441] vs. 446 [415–456] ms) values and pancreatic manganese uptake (30 [28–34] vs. 28 [24–31] mL/100 g of tissue/min) values. There were strong correlations between the scan‐rescan pancreatic native T1 values (mean difference: −3.5 (limits of agreement −47.1 to 40) ms; ICC = 0.84), post‐contrast T1 values (mean difference: 2.1 (limits of agreement: −23.9 to 28.1) ms; ICC = 0.88) and pancreatic manganese uptake (mean difference: −0.72 (limits of agreement: −2.9 to 1.6) mL/100 g of tissue/min; ICC = 0.96).

## 4 DISCUSSION

This is the first prospective clinical study investigating manganese‐enhanced magnetic resonance imaging of the pancreas in people with type 1 diabetes mellitus. We have shown that pancreatic manganese uptake is markedly reduced in people with type 1 diabetes mellitus and that this reduction is directly proportional to plasma C‐peptide concentrations. This technique represents a potential non‐invasive method of quantifying in vivo human pancreatic beta‐cell function and mass with implications for the diagnosis, monitoring of disease progression and assessment of treatment efficacy.

As an analogue of calcium, manganese is readily taken up into cells that use calcium‐dependent processes including hepatic, cardiac and pancreatic cells. Active insulin secretion is highly dependent on intracellular fluxes of calcium. It is therefore unsurprising that manganese‐enhanced magnetic resonance imaging markedly decreases the T1 values of pancreatic beta cells.^20^ There have been previous animal and human studies in diabetes suggesting manganese‐based contrast media have the potential for detecting differences in the functional beta cell mass.^21^, ^22^ However, is manganese uptake specific to the pancreatic beta cells?

Several animal studies have suggested that manganese uptake in the pancreas is specific to beta cells as opposed to exocrine cells or other islet cell populations. Streptozotocin specifically targets pancreatic beta cells, entering through glucose transporter 2, causing beta cell necrosis.^23^ There is a marked decrease in pancreatic manganese uptake in streptozotocin‐treated mice compared to the non‐diabetic mice. Tolbutamide and diazoxide target beta cell adenosine triphosphate‐sensitive potassium channels (K_ATP_), but not exocrine pancreas cells, and selectively activate or inactivate beta cell voltage‐gated calcium channels respectively. Following treatment, pancreatic manganese uptake is increased by tolbutamide and decreased by diazoxide.^24^, ^25^, ^26^ We therefore suggest it is very likely that pancreatic enhancement seen with mangafodipir is predominantly due to manganese uptake in functional beta cells.^15^, ^21^, ^22^, ^27^

For the first time, we provide the proof‐of‐concept that non‐invasive manganese‐enhanced magnetic resonance imaging can be applied in people with type 1 diabetes mellitus to assess beta‐cell function. We have demonstrated that there is a marked suppression of manganese uptake in people with type 1 diabetes mellitus and especially in those with very low plasma C‐peptide concentrations. Indeed, there was a proportional reduction in pancreatic manganese uptake with progressive falls in plasma C‐peptide concentrations. This is consistent with the progressive loss of beta‐cell function and reduced endogenous calcium‐dependent insulin secretion.

Our findings are in keeping with results from pre‐clinical studies exploring manganese enhanced magnetic resonance imaging of the pancreas in rodent models of diabetes.^14^, ^28^ In the murine model of streptozotocin‐induced pancreatic beta‐cell destruction, Meyer et al. reported a 79% reduction in pancreatic manganese uptake which correlated with a 73% loss in beta cell mass.^28^ Similarly, Antkoviak et al. observed a streptozotocin dose‐dependent reduction in pancreatic manganese uptake in a murine model.^22^ In a retrospective analysis of abdominal manganese‐enhanced magnetic resonance imaging, Botsikas et al. found lower enhancement of pancreatic signal in people with type 2 diabetes.^21^ This is likely in keeping with 20%–30% reduction in pancreatic beta‐cell mass that is observed in people with type 2 diabetes. However, this imaging had been performed for other indications, had not been optimised for analysis of the pancreas, and was conducted in the absence of a standardised glucose load.^21^

Our novel imaging method has several important strengths. First, unlike other techniques used for pancreatic beta‐cell imaging, this method does not require isolation or labelling of islet cells.^29^, ^30^ Second, this technique is non‐invasive, does not involve radiation and has excellent observer repeatability and scan‐rescan reproducibility. This would make it an ideal modality for repeated pancreatic imaging which may be required to monitor disease progression as well as response to immunomodulator drugs in clinical trials for type 1 diabetes mellitus. Third, manganese‐enhanced magnetic resonance imaging can assess beta‐cell function dynamically in response to a standardised glucose load and provide a visual assessment of the functional beta cell mass. This could help describe the pattern of beta‐cell loss in the natural history of type 1 diabetes mellitus and in differentiating between different causes of pancreatic insufficiency. This is particularly relevant in the context of diagnostic trials in type 1 diabetes mellitus (such as The Environmental Determinants of Diabetes in the Young (TEDDY) study, NCT00279318) that require invasive pancreatic biopsies which can cause complications and are susceptible to sampling error.^31^ Moreover, in the last two decades, pancreas pathology in type 1 diabetes mellitus has been better characterised and different disease endotypes have been recognised. Recent studies have demonstrated the presence of ‘insulitis’, pathological changes in islet cells in pre‐clinical stages of type 1 diabetes mellitus.^32^ This imaging technique could further contribute to valuable information regarding pre‐clinical stages of type 1 diabetes mellitus. Finally, although blood biomarkers are widely used in clinical practice, there are several important limitations of plasma C‐peptide concentrations. The correlation between plasma C‐peptide concentrations and beta‐cell mass is imperfect and has limitations. The presence of anti‐insulin antibodies that bind both pro‐insulin and C‐peptide can give falsely high C‐peptide concentrations and there is evidence from in vivo studies that plasma C‐peptide concentrations do not correlate with beta‐cell mass in humans.^9^ Similarly, plasma C‐peptide concentrations are challenging to interpret in patients with chronic kidney disease and in the presence of anti‐insulin antibodies that bind both pro‐insulin and C‐peptide.

We acknowledge that our proof‐of‐concept study has several limitations. First, we acknowledge that the patient cohorts and healthy volunteers were not well matched for age, although there was no association between age and pancreatic manganese uptake in either the healthy volunteers or people with type 1 diabetes mellitus. Second, this study comprised of a small sample size and therefore these findings should be further validated in future studies involving larger cohorts. Third, from our study, we cannot be certain that manganese uptake truly reflected functional beta cell mass in the absence of histological correlation and further validation of our findings. Fourth, although we had strong correlations with c‐peptide concentrations, further studies are needed to investigate correlations with insulin sensitivity, dynamic changes in c‐peptide concentrations, and the optimal oral glucose load to best assess pancreatic manganese uptake and assessment of functional beta cell mass. Finally, there are no currently available preparations of manganese‐based contrast medium for widespread clinical use. However, this is likely to change with commercially available preparations anticipated soon.^33^, ^34^

## 5 CONCLUSION

We have demonstrated, for the first time, that manganese‐enhanced magnetic resonance imaging can provide a non‐invasive imaging technique to assess the functional beta‐cell mass in people with type 1 diabetes mellitus. This has important implications for the investigation of the pathophysiology of diabetes, the monitoring of disease progression and the assessment of novel beta‐cell preserving therapeutic interventions.

## FUNDING INFORMATION

SSJ and DEN are supported by the British Heart Foundation (FS/CRTF/20/24087, CH/09/002, RG/05/003, RG/10/9/28286, PG/03/017/15071, RG/16/10/32375, RE/18/5/34216). DEN is the recipient of a Wellcome Trust Senior Investigator Award (WT103782AIA). MCW (FS/ICRF/20/26002) is supported by the British Heart Foundation.

## CONFLICT OF INTEREST STATEMENT

No relevant conflicts of interest to disclose.

## Supporting information


Figure S1.

## Data Availability

The data that support the findings of this study are available from the corresponding author upon reasonable request.
