# Supplementary material for: Non‐invasive imaging of functional pancreatic islet beta‐cell mass in people with type 1 diabetes mellitus
Source: Diabet Med. 2023 Apr 21;40(10):e15111. doi: 10.1111/dme.15111 (PMC10946460; doi:10.1111/dme.15111)
Supplement: Supplementary file 1 — Figure S1. [file DME-40-0-s001.zip › dme15111-sup-0002-FigureS1.docx]

Figure S1. Correlations between pancreatic manganese uptake and baseline plasma C-peptide concentrations in participants with type 1 diabetes mellitus (panel A) and healthy volunteers (panel B).
