# Supplementary figures and images for: Non‐invasive imaging of functional pancreatic islet beta‐cell mass in people with type 1 diabetes mellitus
Source: Diabet Med. 2023 Apr 21;40(10):e15111. doi: 10.1111/dme.15111 (PMC10946460; doi:10.1111/dme.15111)

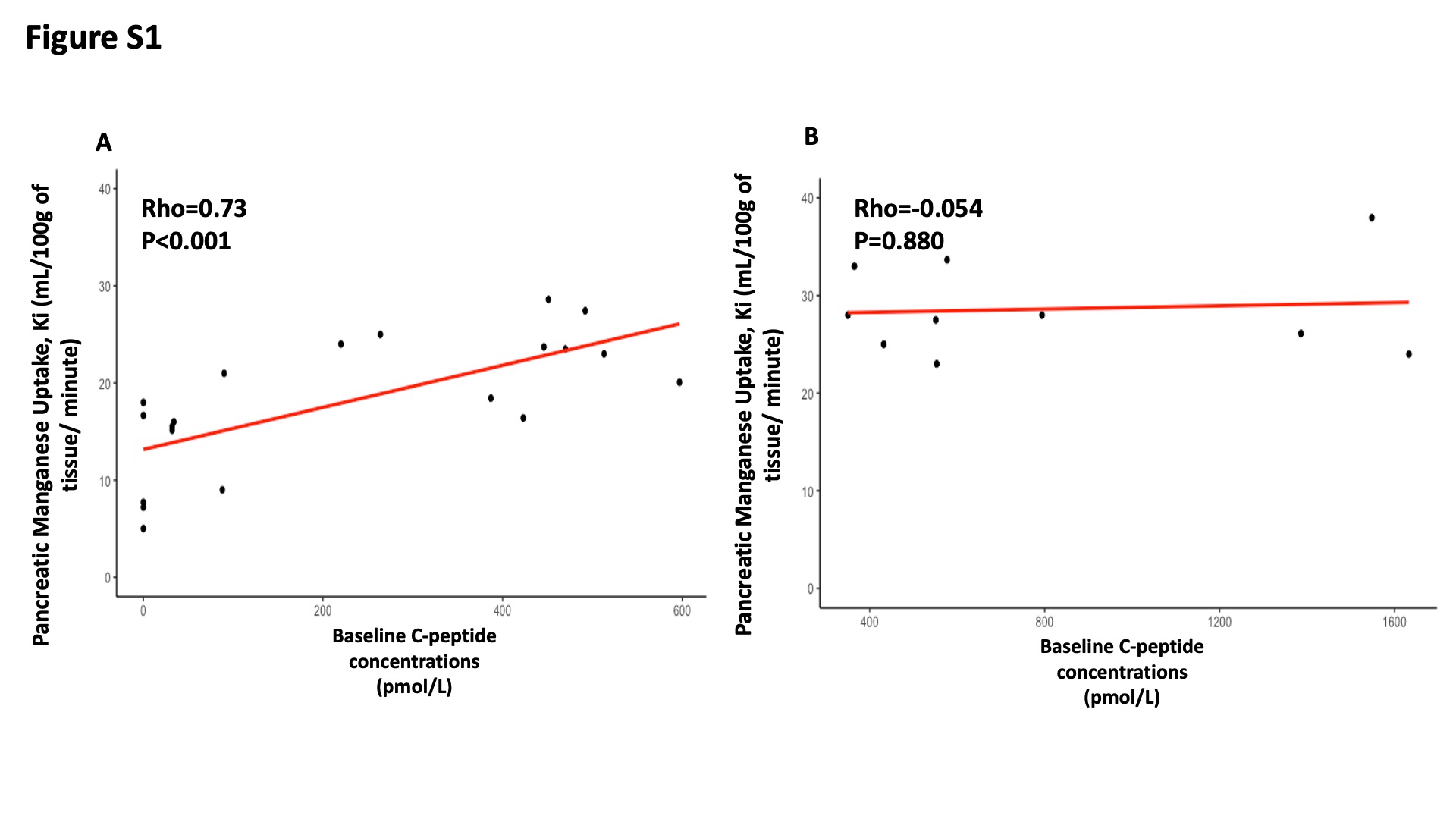

Supplement: Supplementary file 1 — Figure S1. [file DME-40-0-s001.zip › dme15111-sup-0001-FigureS1.jpg]
